# *Candida auris* Outbreak in a Multidisciplinary Hospital in Romania during the Post-Pandemic Era: Potential Solutions and Challenges in Surveillance and Epidemiological Control

**DOI:** 10.3390/antibiotics13040325

**Published:** 2024-04-03

**Authors:** Violeta Melinte, Alexandra Daniela Tudor, Adrian Georgian Bujoi, Maria-Adelina Radu, Maria Cristina Văcăriou, Ioana Miriana Cismaru, Tiberiu Sebastian Holban, Carmen Luminița Mîrzan, Ruxandra Popescu, Robert Cătălin Ciupan, Alin Baciu, Oriana Elena Moraru, Matei Popa-Cherecheanu, Valeriu Gheorghiță

**Affiliations:** 1 Faculty of Medicine, Carol Davila University of Medicine and Pharmacy, 050474 Bucharest, Romania; maria-adelina.cosma@drd.umfcd.ro (M.-A.R.); oriana-elena.moraru@umfcd.ro (O.E.M.); matei.cherecheanu@gmail.com (M.P.-C.); valeriu.gheorghita@umfcd.ro (V.G.); 2 “Agrippa Ionescu” Clinical Emergency Hospital, 011356 Bucharest, Romania; alexandra-daniela.tudor@rez.umfcd.ro (A.D.T.); adrian-georgian.bujoi@rez.umfcd.ro (A.G.B.); vacaroiu.cristina@dcti.ro (M.C.V.); ioana-miriana.cismaru@rez.umfcd.ro (I.M.C.); holban.tiberiu@dcti.ro (T.S.H.); marzanl@dcti.ro (C.L.M.); popescu.ruxandra@dcti.ro (R.P.); ciupan.robert@dcti.ro (R.C.C.); baciu.alin@dcti.ro (A.B.)

**Keywords:** *Candida auris*, hospital outbreak, emerging infections, antimicrobial resistance, healthcare-associated infections, post-pandemic era

## Abstract

*Candida auris* is a newly emerging yeast, which is raising public health concerns due to its outbreak potential, lack of protocols for decontamination and isolation of patients or contacts, increased resistance to common antifungals, and associated high mortality. This research aimed to describe the challenges related to identifying the outbreak, limiting further contamination, and treating affected individuals. We retrospectively analyzed all cases of *C. auris* detected between October 2022 and August 2023, but our investigation focused on a three-month-long outbreak in the department of cardio-vascular surgery and the related intensive care unit. Along with isolated cases in different wards, we identified 13 patients who became infected or colonized in the same area and time, even though the epidemiological link could only be traced in 10 patients, according to the epidemiologic investigation. In conclusion, our study emphasizes the substantial challenge encountered in clinical practice when attempting to diagnose and limit the spread of an outbreak. Therefore, it is crucial to promptly apply contact precaution measures and appropriate environmental cleaning, from the first positive case detected.

## 1. Introduction

In recent decades, multidrug-resistant fungal infections have attracted the attention of the international medical staff [1]. Of all *Candida* species, *Candida auris*, which has a similar level of virulence to *Candida albicans*, but is more difficult to treat [2], became a concern for healthcare workers, due to its capacity to cause outbreaks all over the world [3,4,5,6]. *C. auris*, a yeast which is difficult to identify, can rapidly develop resistance to current antifungal drugs [4,6,7]. Its high level of contagion, the persistence in hospital environment, and the risk of complications in immunocompromised patients make this fungus a public health challenge. In intensive care units it is often associated with mechanical ventilation, invasive procedures, and the presence of invasive medical devices [8].

Since the first identification of *C. auris* in 2009 [9], along with the development of laboratory identification methods, several countries have started to report infections and colonization [10]. In the last decade, microbiological research, based on genetical and molecular biology, has shown the typical features of *C. auris*, highlighting its virulence and epidemiological traces [6].

A small number of samples, retrospectively analyzed in Japan and South Korea, showed that the fungal pathogen was detected before 2009, but it was initially misidentified by conventional methods as *Candida haemulonii*, *Candida famata*, *Rhodotorula glutinis,* or *Saccharomyces cerevisiae* [11]. The SENTRY Antifungal Surveillance Program, conducted between 1997 and 2016, found that of 20,788 *Candida* isolates analyzed retrospectively, consisting of 37 different species, only 6 were found to be *C. auris.* They were all fluconazole-resistant and were associated with hospital-acquired infections. Another finding of this study was that the incidence of *C. glabrata* and *C. parapsilosis* increased as the isolation of *C. albicans* strains declined [12]. Since 2009, hospital outbreaks have been reported in the US, UK, Spain, Italy, Greece, Turkey, Algeria, Kenya, Kuwait, Lebanon, Oman, Pakistan, Venezuela, Colombia, Brazil, Qatar, Saudi Arabia, and India, as well as South Africa [13]. There are five genetically distant clades of *C. auris* identified to date: the South Asian clade (I) detected in India and Pakistan, the East Asian clade (II) detected in Japan, the South African clade (III) identified in South Africa, the South American clade (IV) detected in Venezuela, and the Iranian clade (V) recently discovered in Iran, respectively [4]. Except for the new Iranian and East Asian clades, the other *C. auris* clades have demonstrated the ability to generate outbreaks with invasive infections [14]. However, there is no reported difference in the pathogenicity of the different clades in humans [15].

Studies have found that underlying respiratory diseases and mechanical ventilation in intensive care units are risk factors for *C. auris* infections. Therefore, it has been suggested that the COVID-19 pandemic may have contributed to the appearance of the new outbreaks [16,17]. An epidemiological alert was released by the Pan American Health Organization (PAHO) for outbreaks during the COVID-19 pandemic, including in countries where it had not previously been identified [18,19,20]. In Italy, in February 2020, *C. auris* was identified in a COVID-19 intensive care unit (ICU), with a progressive increase in the number of cases throughout 2020 and 2021. In February 2022, 277 cases were reported in more than eight hospitals in Liguria, with another 11 cases in the Emilia-Romagna region [15]. In Romania, the first cases of *C. auris* infections or colonization were reported at the beginning of 2022 and a study conducted on 40 specimens revealed that they belonged to the South Asian clade (I) [21].

## 2. Results

### 2.1. Case Series Description

**Figure 1:**
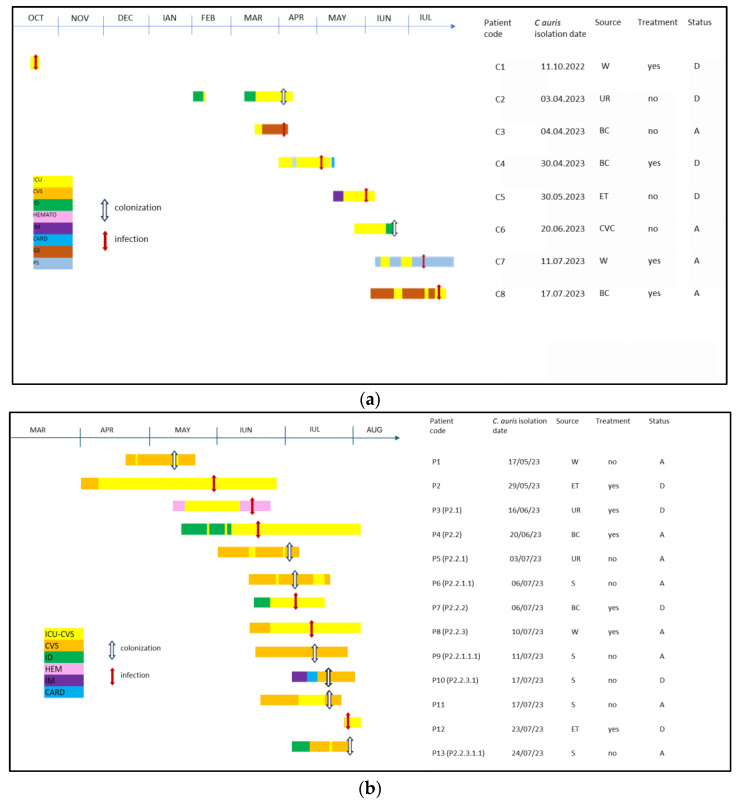
(**a**) *C. auris* dynamics outside the outbreak. C—case; Date dd/mm/yy; W—wound culture; ET—endotracheal tube secretions culture; UR—urine culture; CVC—central venous catheter; BC—blood culture; ICU—intensive care unit; CVS—cardio-vascular surgery ward; ID—infectious diseases ward; HEM—haematology ward; IM—internal medicine ward; CARD—cardiology ward; GS—general surgery ward; PS—plastic surgery ward; D—deceased; A—alive. (**b**) *C. auris* outbreak dynamics. P—patient; Date dd/mm/yy; W—wound culture; ET—endotracheal tube secretions culture; UR—urine culture; BC—blood culture; S—skin carrier; ICU-CVS—cardiovascular surgery associated intensive care unit; CVS—cardio-vascular surgery ward; ID—infectious diseases ward; HEM—haematology ward; IM—internal medicine ward; CARD—cardiology ward; D—deceased; A—alive.

**Figure 2:**
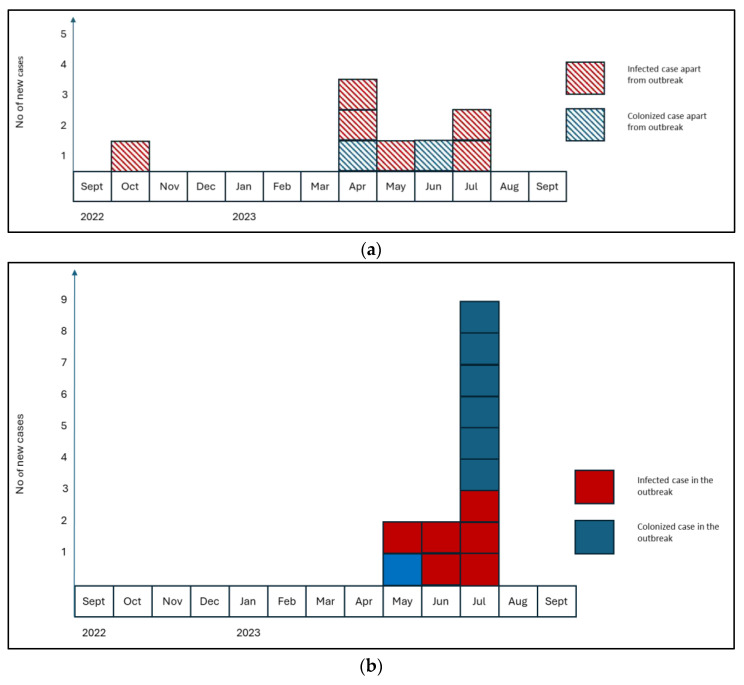
(**a**) Histogram of *C. auris* onset of illness, apart from outbreak. (**b**) Histogram of *C. auris* onset of illness in the outbreak.

We present a series of cases of *C. auris* infections and/or colonization, reported from October 2022 to July 2023 in a public university hospital in Bucharest, mainly in the cardiovascular surgery (CVS)-associated intensive care unit (ICU). Patients who underwent surgical procedures were screened for Methicillin-resistant *Staphylococcus aureus* and received perioperative antibiotic prophylaxis [22]. Of the 21 reported cases with *C. auris* during this period, some were isolated cases—in different departments—and others met the case definition for an outbreak diagnosed during the hospitalization (Figure 1a,b and Figure 2a,b).

**Figure 3:**
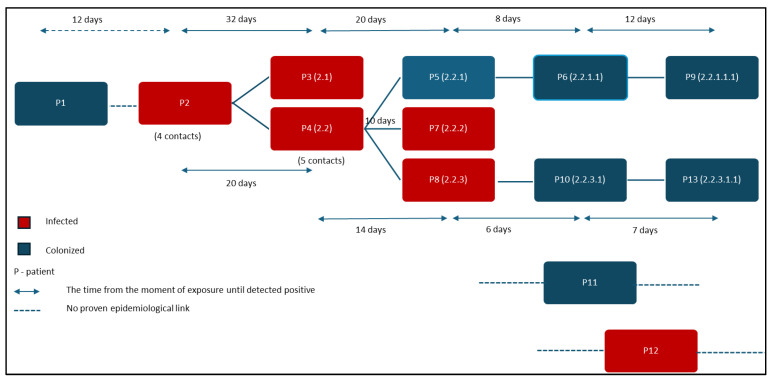
Epidemiological link in *C. auris* outbreak.

According to national regulations, only 13 cases were identified as part of the outbreak, sharing the same area of the hospital. Each patient who tested positive had a close connection to ICU-CVS and shared a pathogenic profile as cardio-vascular disease afflicted patients. When two or more strains of *C. auris* were detected in the same period and on same ward we declared an outbreak [23]. Among these cases, only 10 patients had an evident epidemiological link from sharing the same room, and these patients were mostly in the ICU department (Figure 3).

### 2.2. Outbreak Description

Positive patients were labeled based on the chronological order of their detection, from P1 to P13 (Figure 1b). Once a link between cases was found, we further categorized them through filiation, keeping the index case number followed by a new case number (Figure 3).

Patient number one (P1) admitted to the CVS department, an unlikely index case, had a strain of *C. auris* isolated from the postoperative wound without indication for treatment, and was considered a colonization case. Subsequently, the index case of the declared outbreak, who had no known contact with P1, but this is difficult to rule out, was a 75-year-old patient (P2) who was admitted to the CVS department. He underwent cardiac surgery on the seventh day of hospitalization and rested in the CVS-ICU with respiratory sepsis, mechanical ventilation, and underlying cardiovascular disease. *C. auris* was isolated from his tracheal aspirate on day 52 after surgery. Of the four contacts of patient P2 that were followed over time, two were subsequently diagnosed with *C. auris* infection. The first (P2.1) was detected with a *C. auris* urinary tract infection (UTI) after 32 days of sharing the same room, and the second (P2.2) was diagnosed with a *C. auris* systemic infection after 20 days, respectively. Of the five contacts of patient P2.2, three became positive for *C. auris* in the days following exposure: urinary tract colonization after 20 days (P2.2.1), systemic infection after 10 days (P2.2.2), and superficial surgical site infection after 14 days (P2.2.3). The contact of patient P2.2.1 became positive after 8 days (P2.2.1.1), and subsequently, his contact became positive after 12 days (P2.2.1.1.1), with both cases having skin colonization with a favorable outcome. In the same scenario was the contact of patient P2.2.3 who became positive after 6 days (P2.2.3.1), and subsequently, his contact became positive after 7 days (P2.2.3.1.1), both cases having skin colonization with different outcomes, depending on the underlying comorbidities (Figure 3).

Patient P11 was not in contact with any other *C. auris* positive patients. However, the detection of *C. auris* on the skin after 21 days of hospitalization in the same department reflects the high risk of transmission, despite the prevention and control measures imposed at the detection of the outbreak.

Patient P12, transferred from another hospital in a critical condition requiring mechanical ventilation from admission, and was found to be positive for *C. auris* in a tracheal aspirate specimen collected on the third day after the admission. It is not clear whether the respiratory detection of *C. auris* in this patient was secondary to the infection acquired in our hospital.

### 2.3. Antifungal Susceptibility Profile

Using the CDC tentative MIC breakpoints for the susceptibility interpretation [24], we found that all isolates were resistant to amphotericin B with an MIC ranging between 4 μg/mL and more than 16 μg/mL. A single isolate was susceptible to fluconazole, and only one different strain was pan-drug resistant, considering that it was tested against all antifungals except for fluconazole (Table 1).

### 2.4. Epidemiological Measures for Outbreak Control and Limitation

Appropriate procedures for cleaning, environmental disinfection, and sterilization of medical devices, along with standard precautions and those addressed to the transmission pathway, are the basis for preventing and limiting the transmission of any pathogen in a healthcare facility. According to the international and national guidelines, once a strain of *C. auris* was detected, the identified patients were isolated or grouped in a single room [15,25]. Dedicated nursing staff and medical equipment were established for colonized or infected patients. All infected materials were collected before leaving the room to minimize further transmission.

Instructions on a hand hygiene protocol, contact precautions, and monitoring the appropriate implementation of environmental cleaning for all healthcare workers have been resumed. Family members and visitors were also notified and advised to wear gloves and gowns.

Periodic cleaning of surfaces and medical equipment, as well as terminal cleaning and disinfection of the rooms with chlorine-based disinfectants and hydrogen peroxide, were carried out. Close contacts were screened by swabbing the axilla and groin, throat, (surgical site) wounds, catheter exit sites, and urine, from four patients were identified as skin carriers (Figure 3). Although screening of health care personnel and monitoring of the hospital environment are regularly recommended as part of the prevention and control strategy of healthcare-associated infections, the extent to which screening of medical staff is beneficial in the event of a *C. auris* outbreak remains to be demonstrated.

All cases were reported to the National Institute of Public Health. At discharge, the status of infection or colonization with *C. auris* was mentioned in the medical documents for all patients.

## 3. Discussions

### 3.1. C. auris Epidemiological Features

A positive culture for *C. auris* may reflect either colonization or infection affecting one or more sites and has the potential to lead to severe, life-threatening invasive infections, depending on underlying medical conditions [26]. *C. auris* colonizes the skin, usually in the inguinal region, the axilla, the nostrils, the urinary and respiratory tracts, and, though more uncommon, the intestinal mucosa [27,28]. In 2021, Rossow et al. conducted a study that demonstrated a 10-fold increased risk of colonization for patients on mechanical respiratory support or those who had received treatment with carbapenems or fluconazole within the previous 90 days [29].

In addition to candidemia, *C. auris* is also involved in complicated pleural effusions, pericarditis and ventriculitis, intra-abdominal infections, osteomyelitis, meningitis, and mastoiditis [13,28,30].

Until 2021 there were no *Candida auris* strains reported from Romania to the European Center for Disease Prevention and Control (ECDC) [10]. The first published study refers to 40 strains obtained from three hospitals in Bucharest between January and August 2022 [21]. The comparatively low number of documented cases for Romania, contrasting with Italy or Greece, most likely indicates the underdiagnosing and the lack of screening for this pathogen in patients with evident risk factors.

In our study, nine patients out of the twenty-one identified had colonization of *C. auris*: six on the skin, two in the urinary tract, and one on a central venous catheter (CVC). Twelve of the patients were deemed to be infected with the following principal causes identified: urinary tract infections (*n* = 1), respiratory disorders (*n* = 3), infected wounds (*n* = 3), and systemic diseases (*n* = 5). Three of the five patients with systemic infections diagnosed with positive blood cultures survived, while all patients with respiratory tract infections (*n* = 3) died (Figure 2).

In the ICU, mortality might be more a consequence of the underlying disease and its complications than *C. auris* infection per se [31,32,33]. The true correlation between mortality risk and *C. auris* infection is difficult to determine due to the severe medical conditions of the patients [34,35]. Some studies have ranked mortality according to the clades to which the isolated strains belong, the highest being for South America clades (96%), followed by Asian clades (80%), South African clades (45%), and East Asian clades (44%) [36]. In our study, regardless of detection site, nine of the twenty-one patients died (42.8%). Epidemiological data suggest that candidemia is usually associated with the South American and South Asian clades, while the East Asian clade is frequently associated with otitis externa, and the South African clade is responsible for most of the colonizations and urinary tract infections [37].

As an opportunistic pathogen, *C. auris* infections share some common risk factors with the rest of the *Candida* family. It is also able to colonize the patient without infecting, thus it is a major risk factor for fungal outbreaks due to its persistence and easy transmission [38,39]. Immunosuppression induced by immunosuppressive treatments, including corticosteroids, or acquired after specific medical conditions such as organ transplantation [39], bone marrow transplantation, prolonged administration of broad-spectrum antibiotics or antifungals, and neutropenia are important risk factors [36]. Underlying diseases such as diabetes, malignancies, HIV infection, chronic kidney and respiratory diseases, and hemodialysis are also risk factors for colonization and infection with *C. auris* [40,41,42,43,44]. Invasive procedures such as mechanical ventilation or minimally invasive techniques such as the insertion of central venous catheters, urinary catheters, peripheral intravascular lines, or drain tubes may predispose patients to colonization and infection with *C. auris*, with a greater risk of invasive infection [17,42,45,46]. Most of these risk factors are present in patients with immunosuppressive conditions and in cases of prolonged ICU hospitalization [43,47]. The more invasive the *C. auris* infection, the higher the risk of mortality [14]. All our subjects were intensive care patients from both medical and surgical wards with a long length of hospital stay. However, not all could be epidemiologically involved in the reported outbreak due to a known lack of close contact (e.g, P1 and P11) or possible HAI from another medical facility in the case of P12. This may support the high capacity of *C. auris* to spread and survive on surfaces for long periods of time [15,19,46]. Further molecular investigations could help to conform clade I inclusion or cluster filiation, as we showed epidemiologically. Also, these may shed light on the correlations, or whether there are any, between the genotype and virulence or outbreak potential.

*C. auris* has an unexpected ability to resist surface disinfection procedures compared to the rest of the *Candida* species [48,49]. Widespread contamination of objects and surfaces around a patient colonized with *C. auris* has been demonstrated. It was observed that 75% of the samples collected from the living room surfaces of a colonized or infected patient were positive in molecular biology tests, respectively, and 25% were positive in a culture [50]. *C. auris* has a high potential for transmission, which occurs through direct contact or through colonized surfaces and objects that have been in contact with an infected patient [46]. The most incriminated objects responsible for transmission were blood pressure cuffs, thermometers, bells, and textile cord [51,52,53], and the most frequently colonized sites were the axilla, although for how long the patient may remain colonized has not yet been investigated [13].

Screening is performed by swabbing the patient’s axilla and groin bilaterally, followed by inoculation of the samples in Sabouraud broth, which contains dulcitol and 10% salt [54]. A characteristic of this fungus is its good growth at temperatures between 40 and 42 °C [55]. Detection of *C. auris* is complicated because other closely related fungi have similar patterns of assimilation and fermentation properties. Many of the earliest isolates of *Candida auris* were misidentified as *Rhodotorula glutinis*, *Saccharomyces cerevisiae*, or *Candida sake* by the API 20C AUX, AP ID 32C systems or as *Candida haemulonii* by the Vitek 2 system [56,57,58]. For several decades, chromogenic methods of *Candida* species identification have played a major role in diagnosis [59]. However, to differentiate *C. auris* from the other *Candida* species, additional techniques are needed. Given that *C. auris* does not possess pseudohyphae or germ tubes [55], after chromogenic identification it is necessary to confirm the infection either by using DNA sequencing or matrix-assisted laser desorption ionization-time of flight mass spectrometry (MALDI-TOF MS) [60].

More studies are necessary for a better understanding of the transmission dynamics of this fungus. We know little about the contact characteristics associated with cross-transmission such as type of contact, duration of the contact, and the minimum threshold in the case of contact in which there is no significant risk of transmission. In addition, there is still no consensus regarding the duration of surveillance after contact, how frequently we should test the contacts, or after how many negative tests it is safe to discontinue the isolation. Further questions remain such as should we report negative tested contacts? Could topical skin decolonization be efficient in lowering the risk of transmission? Is screening for *C. auris* as indicated recommended for other pathogens such as extended-spectrum beta-lactamase or carbapenem-producing Gram-negative bacteria or methicillin-resistant *Staphylococcus aureus*?

### 3.2. Present Therapies and New Directions

The most used antifungal agents for the treatment of *Candida* infections include azoles and echinocandins such as caspofungin and micafungin. However, *C. auris* isolates frequently show resistance to fluconazole, while their resistance to other antifungal agents shows a greater variability.

Therapeutic guidelines for the management of *C. auris* recommend the initiation of echinocandin monotherapy as an empiric treatment before the results of susceptibility testing are available. This approach relies on the known prevalence of the resistance profile [61,62,63]. Despite emerging reports of echinocandin and pan-resistant isolates, in regions where most strains remain susceptible, the use of echinocandin as a primary treatment is considered reasonable [62]. However, patients should be closely monitored for clinical and microbiological responses by performing cultures and susceptibility testing, as the organism can develop resistance rapidly even during treatment [64].

In our study, for patients with clinical and biological signs that were highly suggestive of an active infection, antifungal therapy was recommended and included echinocandins according to the patients susceptibility profile (Figure 1a,b). Two infected patients (C3 and C5) did not receive antifungals because one of them died and the other one was transferred before the results were available (Figure 1a).

There is little evidence regarding the most appropriate therapy for pan-resistant strains, which express resistance to all three major classes of antifungal drugs, such as echinocandins, amphotericin B, and the azoles [7]. In vivo studies have shown the inhibition of pan-resistant strains using combinations of two antifungal drugs at fixed concentrations. Favorable responses have been achieved from combinations of flucytosine with amphotericin B, azoles, or echinocandins [65]. Additionally, in vitro evidence supports the use of echinocandin combination therapy, such as caspofungin in combination with posaconazole [66] or anidulafungin in combination with manogepix or flucytosine [67]. A new echinocandin, rezafungin, which is currently in phase 3 trials [68,69] also shows promise based on in vitro investigations against echinocandin-resistant *C. auris* subgroups. Fosmanogepix, a pioneering antifungal with a unique mechanism of action, available in intravenous and oral forms, has shown potential activity in both in vitro and phase 2 studies [70,71]. For persistent and recurrent *C. auris* bloodstream infections, micafungin combination therapies appear promising based on animal studies and in vitro evaluations [62,63,64,65,66,67,68,69,70,71,72,73].

The CDC recommends screening patients with recent overnight stays in healthcare facilities outside the United States and those who have infections or colonization with carbapenemase-producing Gram-negative bacteria. Screening should include the axilla and groin and additional sites as clinically indicated or where previous infections have been detected. Reassessment should occur at 1-to-3-month intervals. At least two evaluations at 1-week intervals are required for deisolation, with negative results after the discontinuation of antifungal treatment [74]. For the same purpose, the CDC recommends using standard single room contact precautions with a gown and gloves. Practicing hand hygiene with alcohol- or water-based hand sanitizer and soap if hands are visibly soiled and retraining staff should also be considered. Roommates with whom the index patient has stayed in the past month, or those with whom they were in contact for at least 3 days, and those with mechanical ventilation or other higher levels of care should also be screened, and bilateral screening for axillary and inguinal colonization is required [74]. Treating colonized patients without any evidence of infection is not recommended, and decolonization protocols do not yet exist [75]. Prophylactic measures remain the main tool for avoiding or stopping *C. auris* outbreaks. For final room decontamination, the ECDC recommends the use of chlorine-based disinfectants, hydrogen peroxide, or other documented fungicidal agents. Avoiding quaternary ammonium compounds is recommended. Preferably, the use of disposable equipment or equipment dedicated to patients with *C. auris* is recommended [75].

Our study has some limitations, such as the small number of cases included in the analysis, the absence of sequencing data and the establishment of lineage, and the monitoring of the clinical and microbiological evolution of colonized or infected patients was only carried out during the hospitalization period.

## 4. Materials and Methods

We present a retrospective epidemiological analysis of 21 nonduplicate clinical specimens of *C. auris* isolated from individual patients admitted to different wards of the “Agrippa Ionescu” Clinical Emergency Hospital, Bucharest, Romania, between October 2022 and July 2023. Patients were diagnosed with *C. auris* bloodstream infections (*n* = 5), respiratory tract infections (*n* = 3), and colonization or asymptomatic infection in different sites such as skin, urine, wounds, catheters. Of these, we focused on 13 strains which were considered as outbreaks because they were found in the same area and time and required similar surgical interventions.

All isolates were cultivated using Sabouraud Gentamicin Chloramphenicol 2 Agar and CHROMID^®^ Candida Agar (BioMérieux, Marcy-l’Étoile, France) and grown for 48 h at 30˚C. The species-level identification was carried out using fluorescent-based technology (Advance Colorimetry^™^—VITEK 2^®^ COMPACT system; BioMérieux, Marcy-l’Étoile, France) and VITEK 2^®^ PC Software v9. The growth of microorganisms in blood cultures was screened by the BacT/Alert Microbial Detection System (Organon Teknika). This is an automated test system capable of incubating, agitating, and continuously monitoring aerobic and anaerobic media inoculated with specimens from patients suspected of having bacteremia.

Antifungal susceptibility testing for four antifungal agents (i.e., caspofungin, micafungin, fluconazole, amphotericin B) was performed according to the CLSI M27-A3 guidelines, using the broth microdilution minimum inhibitory concentrating (MIC) technique (VITEK 2^®^ COMPACT system; BioMérieux, Marcy-l’Étoile, France). As *C. auris*-specific susceptibility breakpoints have not yet been established, tentative breakpoints proposed by the Centers for Disease Control and Prevention (CDC) or previously adopted by other studies were used [24]. Thus, resistance breakpoints were defined as follows: caspofungin at ≥2 pg/mL, micafungin at ≥4 pg/mL, fluconazole at ≥32 pg/mL, amphotericin B at ≥2 pg/mL.

## 5. Conclusions

Our study highlights the substantial challenges encountered in clinical practice when attempting to diagnose and limit the spread of an outbreak of *C. auris*, which are largely explained by the increased transmissibility and treatment difficulties. Therefore, it is crucial to promptly apply contact precaution measures and appropriate environmental cleaning following the detection of the first positive case.

The use of standard prevention procedures is still the only epidemiologically effective strategy that can decrease the morbidity and mortality rates associated with a *C. auris* outbreak, as we still lack a specific action protocol. This research reveals a potentially increased circulation of *C. auris* in hospital environments, which raises the question of the feasibility of implementing specific screening for vulnerable patients.

More studies are necessary for a better understanding of the transmission dynamics of this fungus, for better outcomes with respect to efficiently containing outbreaks, and for the development of more effective antifungal therapies.

## Figures and Tables

**Table 1:** In vitro antifungal susceptibility profiles of characterized *C. auris* isolates (*n* = 21). (MICs in mg/L).

MICs	CAS	MIC	FLU	AMB	
Breakpoints *	≥2	≥4	≥32	≥2	Label
Pt. 1	≥8	≥8	nt	8	C1
Pt. 2	0.25	0.06	32	8	C2
Pt. 3	0.25	0.12	32	0.5	C3
Pt. 4	0.25	0.12	16	8	C4
Pt. 5	0.25	0.12	32	4	C5
Pt. 6	0.25	nt	16	8	C6
Pt. 7	0.25	0.12	32	4	C7
Pt. 8	0.25	0.12	32	≥16	C8
Pt. 9	0.25	0.12	32	8	P1
Pt. 10	0.25	0.12	32	8	P2
Pt. 11	0.25	0.12	32	8	P3
Pt. 12	0.25	0.12	32	≥16	P4
Pt. 13	0.25	0.12	32	8	P5
Pt. 14	0.25	0.5	32	8	P6
Pt. 15	0.25	0.5	32	8	P7
Pt. 16	0.25	nt	32	8	P8
Pt. 17	0.25	0.12	32	8	P9
Pt. 18	0.25	0.12	32	8	P10
Pt. 19	0.25	0.12	32	≥16	P11
Pt. 20	0.25	0.12	16	≥16	P12
Pt. 21	0.25	0.12	32	8	P13

Abbreviations: AMB—Amphotericin B, FLU—Fluconazole, MIC—Micafungin, CAS—Caspofungin; nt—not tested; C1–8—cases apart from outbreak; P1–13—patients in the outbreak * CDC tentative breakpoints [24].

## Data Availability

Data are contained within the article.
